# Evidence of Differences in Covariation Among Root Traits Across Plant Growth Forms, Mycorrhizal Types, and Biomes

**DOI:** 10.3389/fpls.2021.785589

**Published:** 2022-01-28

**Authors:** Nannan An, Nan Lu, Bojie Fu, Weiliang Chen, Maierdang Keyimu, Mengyu Wang

**Affiliations:** ^1^State Key Laboratory of Urban and Regional Ecology, Research Center for Eco-Environmental Sciences, Chinese Academy of Sciences (CAS), Beijing, China; ^2^University of Chinese Academy of Sciences, Beijing, China; ^3^Faculty of Geographical Science, Beijing Normal University, Beijing, China

**Keywords:** belowground strategy, fine-root trait, mycorrhizal symbiosis, phylogeny, plant growth form, root economics spectrum

## Abstract

Fine roots play an important role in plant ecological strategies, adaptation to environmental constraints, and ecosystem functions. Covariation among root traits influence the physiological and ecological processes of plants and ecosystems. Root trait covariation in multiple dimensions at the global scale has been broadly discussed. How fine-root traits covary at the regional scale and whether the covariation is generalizable across plant growth forms, mycorrhizal types, and biomes are largely unknown. Here, we collected six key traits – namely root diameter (RD), specific root length (SRL), root tissue density (RTD), root C content (RCC), root N content (RNC), and root C:N ratio (RCN) – of first- and second-order roots of 306 species from 94 sampling sites across China. We examined the covariation in root traits among different plant growth forms, mycorrhizal types, and biomes using the phylogenetic principal component analysis (pPCA). Three independent dimensions of the covariation in root traits were identified, accounting for 39.0, 26.1, and 20.2% of the total variation, respectively. The first dimension was represented by SRL, RNC, RTD, and RCN, which was in line with the root economics spectrum (RES). The second dimension described a negative relationship between RD and SRL, and the third dimension was represented by RCC. These three main principal components were mainly influenced by biome and mycorrhizal type. Herbaceous and ectomycorrhizal species showed a more consistent pattern with the RES, in which RD, RTD, and RCN were negatively correlated with SRL and RNC within the first axis compared with woody and arbuscular mycorrhizal species, respectively. Our results highlight the roles of plant growth form, mycorrhizal type, and biome in shaping root trait covariation, suggesting that root trait relationships in specific regions may not be generalized from global-scale analyses.

## Introduction

Fine roots play a multifaceted role in plant resource acquisition, adaptation to environmental changes (Diaz et al., 2004; McCormack et al., 2014), and ecosystem processes (e.g., carbon and nutrient cycling, net primary production, and soil formation) (Cornwell et al., 2008; De Deyn et al., 2008; McCormack et al., 2015). The impact of roots on plant growth and ecosystem processes largely depends on the covariation among the morphological, chemical, and physiological traits of fine roots (Eviner and Chapin Iii, 2003; Bardgett et al., 2014). Assessing how root traits are interrelated and whether the interrelationships are broadly generalizable can help us better understand belowground resource acquisition strategies and ecosystem functions under environmental change.

Numerous studies have corroborated the generality of the leaf economics spectrum (LES) across plant growth forms, biomes, and spatial scales (Reich et al., 1992, 1999; Wright et al., 2004; Shipley et al., 2006; Hu et al., 2015). However, our understanding of the root economics spectrum (RES) – that demonstrates the unidimensional acquisitive–conservative gradient – lags far behind that of the LES, because conceptual and methodological challenges associated with root traits have impeded data standardization and integration within traits (Delory et al., 2017; Guerrero-Ramírez et al., 2021). The RES hypothesis proposes that roots with an acquisitive strategy are characterized by a thinner diameter, higher specific root length (SRL), and higher root N content (RNC). In contrast, roots with a conservative strategy are represented by a thicker diameter, lower SRL and RNC, and higher root C:N ratio (RCN) (Freschet et al., 2010; Reich, 2014; Roumet et al., 2016). The notion of one-dimensional RES has been supported by some regional studies (Roumet et al., 2016; de la Riva et al., 2018). For example, de la Riva et al. (2021a) found that SRL and specific root area (SRA) were negatively related to root tissue density (RTD) and root dry matter content in Mediterranean vegetation.

However, recent global studies have provided growing evidence for the multiple dimensions of root trait covariation (Bergmann et al., 2020; Weigelt et al., 2021). Two or more independent gradients were found to represent different facets of root trait syndromes (Weemstra et al., 2016). Bergmann et al. (2020) demonstrated two dimensions of the root economics space at the global scale. One was defined as the collaboration gradient represented by a trade-off between root diameter (RD) and SRL, and the other was defined as the conservation gradient represented by the negative relationship between RTD and RNC. This multidimensional trait pattern allows roots to enhance resource uptake from soils through either the construction of thinner fine roots or the development of high mycorrhizal dependency (Bergmann et al., 2020; Weigelt et al., 2021), reflecting the root trait diversity and adaptation to different environments.

The discrepancy of root trait covariation between global and regional studies may be related to the bias in the geographical coverage of the global root trait database (Guerrero-Ramírez et al., 2021) and the scale-dependence of root trait relationships (de la Riva et al., 2016b; Messier et al., 2017). These factors are important because selective pressures vary across spatial scales (Albert et al., 2010; Liu et al., 2010), and different traits have different sensitivities to the same pressures (Messier et al., 2010; Shiklomanov et al., 2020). For example, recent studies demonstrated that root trait-environment relationships and trait coordination became less clear with decreasing spatial scales (de la Riva et al., 2016b; Liu H. et al., 2019). Moreover, regional studies about the covariation among root traits have also reported contradictory results. For the same set of root traits – namely RD, SRL, SRA, RTD, RNC, root C content (RCC), RCN, stele diameter, and cortex thickness – Liu C. et al. (2019) reported two primary dimensions of trait covariation in the subtropical forests of China, whereas Zhou et al. (2018) demonstrated three main dimensions in the temperate steppes of China. These inconsistencies in results among the regional studies may be related to multiple factors, including the differences in the selection of root traits defining the trait coordination and trade-offs (de la Riva et al., 2021a), species composition (e.g., plant growth form and phylogenetic group) (Wang et al., 2018a; Weigelt et al., 2021), mycorrhizal association type (Comas et al., 2014; Akatsuki and Makita, 2020), and biome type (Wang et al., 2018b; Valverde-Barrantes et al., 2021).

Plant growth form is an important factor shaping root trait syndromes and may influence the root form and function (Freschet et al., 2017; Valverde-Barrantes et al., 2017). For example, Roumet et al. (2016) suggested that the root traits of herbaceous species exhibited a more consistent pattern with the RES hypothesis than those of woody species from temperate, Mediterranean, and tropical biomes in France and China. The potential role of mycorrhizal symbiosis in plant resource uptake strategies is also gaining concerns (Weemstra et al., 2016; Laliberte, 2017; Gao et al., 2021). Ding et al. (2020) found the two dimensions of root trait covariation (that is, RD and SRL vs root nutrients and RTD) in ectomycorrhizal (ECM) species in the eastern Tibetan Plateau and such pattern has been found for arbuscular mycorrhizal (AM) species in a temperate rain forest in New Zealand (Kramer-Walter et al., 2016). To date, there has been no consensus regarding the covariation among root traits across plant growth forms (Adams et al., 2013; Sun et al., 2016) or types of mycorrhizal association (Cheng et al., 2016; Kong et al., 2019).

Another knowledge gap in root ecology involves a comparison of root trait covariation across biomes, as plants adapted to different environmental conditions may exhibit diverse root trait syndromes (Ma et al., 2018; Valverde-Barrantes et al., 2021). According to the RES hypothesis, SRL is expected to be negatively associated with RTD, reflecting the trade-off between root lifespan and construction cost (Roumet et al., 2016; Weemstra et al., 2016). However, this relationship is not observed in all biomes; some studies have described a significant relationship in Mediterranean forests and shrublands and temperate grasslands (de la Riva et al., 2018; Zhou et al., 2018), whereas others studies in temperate rain forests, subtropical forests, and temperate forests have not (Chen et al., 2013; Kong et al., 2014; Kramer-Walter et al., 2016). Therefore, there is a need for comparative studies on the covariation of root traits among multiple biomes (Laughlin et al., 2021).

China is one of the richest countries in terms of plant diversity with ancient origin and complex composition of the flora with varied types of plant-mycorrhizal symbiosis (Liu et al., 2003; Huang, 2011). It contains multiple biome types, ranging from boreal forests to tropical forests (north to south), and from savannas to alpine tundra (northeast to west and southwest) (Ni, 2001). This variety provides an ideal opportunity to test the root trait covariation and their drivers by including all of the abovementioned factors and to provide an integrated understanding of root trait covariation of the region. We compiled a dataset of six key fine-root traits of 306 species from 94 sampling sites across China. The six root traits were RD, SRL, RTD, RCC, RNC, and RCN, which are widely studied traits related to the RES (Weemstra et al., 2016; Liu C. et al., 2019). These traits are closely associated with plant growth rate, construction cost, and lifespan (Comas and Eissenstat, 2004; Roumet et al., 2016), and reflect the trade-offs between resource acquisition and resource conservation (Reich, 2014). Our objectives were to (1) examine the covariation among the six root traits; and (2) test the generality of the covariation among root traits across plant growth forms, mycorrhizal types, and biomes.

## Materials and Methods

### Data Collection

We collected the root trait data of plants in China from the global Fine-Root Ecology Database (FRED^1^) (Iversen et al., 2017) and the published literature (listed in Note 1 in Supplementary Material). We focused on first- and second-order roots that are defined by branching order, where the most distal roots are numbered as first order and where second-order roots begin at the junction of two first-order roots (Pregitzer et al., 2002). The first- and second-order roots generally belong to absorptive roots (Guo et al., 2008; Freschet and Roumet, 2017), which have high physiological activity and resource acquisition efficiency (McCormack et al., 2015). Thus, the studies that used diameter-based fine roots (e.g., ≤1 mm, ≤2 mm, and ≤0.5 mm) and absorptive roots without a clear root branching order were excluded. To ensure data quality and homogeneity, root trait data were obtained according to the following criteria: (1) studies conducted in fields were included and those conducted in croplands, aquatic ecosystems, greenhouses, and laboratories were excluded, in order to minimize the effects of management disturbance; (2) root samples were collected from mature and healthy plant individuals to minimize the effects of ontogeny (Alvarez-Flores et al., 2014); (3) root samples were collected from live roots and data from dead roots were excluded to reduce the confounding effects of root vitality; and (4) root samples were collected from native species and non-native species were excluded. When a species occurred at multiple sampling sites, all site–species trait values were recorded.

Species name and taxonomic nomenclature were standardized and corrected according to the Plant List^2^ using the “plantlist” package. A total of 407 site × species observations of 306 species from 72 families and 174 genera were collected. The species were classified into seven phylogenetic clades according to APG IV classification (APG, 2016): gymnosperms, chloranthales, monocots, magnoliids, basal eudicots, asterids, and rosids. Basal eudicots include species that are not included in the clades of rosids and asterids. The information on plant growth form (herbaceous and woody) was obtained from the original literature and the TRY–Categorical Traits Dataset^3^ (Kattge et al., 2011). Our dataset included 53 herbaceous and 253 woody species, respectively.

The information regarding the type of mycorrhizal association was collected and corrected according to the original literature and previously published databases (Wang and Qiu, 2006; Hempel et al., 2013; Phillips et al., 2013; Valverde-Barrantes et al., 2017; Guerrero-Ramírez et al., 2021). In this study, mycorrhizal types were classified into five groups based on the Global root traits (GRooT) database (Guerrero-Ramírez et al., 2021): AM (221), ECM (68), ericoid mycorrhizal (ERM, 2), AM-ECM (8), and AM-non-mycorrhizal (AM-NM, 7).

The species included in this study accounted for almost all major biomes in China, including forests, grasslands, and alpine tundra. The forests were divided into four biome types: tropical forest (latitude < 23.5°N), subtropical forest (latitude 23.5°–34°N), temperate forest (latitude 34°–50°N), and boreal forest (latitude > 50°N). Species with multiple biome entries were categorized into the biome in which they had the most observations. In this dataset, the tropical forest biome contained 31 species, subtropical forest 144 species, temperate forest 77 species, boreal forest 10 species, grassland 15 species, and the alpine tundra 29 species.

### Construction of the Phylogenetic Tree

The phylogenetic tree was constructed based on the comprehensive angiosperm species-level phylogeny of Zanne et al. (2014) updated by Qian and Jin (2016). The phylogenetic tree was constructed using the “S.PhyloMaker” function in the “phytools” package (Revell, 2012). All 306 species were included in this phylogenetic tree.

### Data Analyses

Before data analyses, all root trait data were log_10_-transformed to meet the assumption of normality. In this study, all data analyses were conducted at the species level, species mean trait values were thus used. We only focus on interspecific trait variation in our analysis.

Blomberg’s *K* (Blomberg et al., 2003) and Pagel’s λ (Pagel, 1999) values were calculated to assess the strength of phylogenetic signals of root traits using the “phytools” package (Revell, 2012). Blomberg’s *K* values > 1 suggest higher phylogenetic conservatism than expected owing to the Brownian motion, and values <1 suggest weaker phylogenetic conservatism. Pagel’s λ values close to 0 indicate phylogenetic independence, and values closer to 1 indicate that the trait distribution perfectly complies with Brownian motion (Münkemüller et al., 2012).

Correlations among root traits were examined using Pearson’s correlation analysis using the “Hmisc” package and phylogenetic independent contrasts (PICs) that account for the phylogenetic relatedness among species using the “picante” package (Felsenstein, 1985). Linear and non-linear regressions were also performed to evaluate the pairwise relationships among root traits. These analyses were conducted for all species and within different plant growth forms, mycorrhizal types, and biomes.

Considering the phylogenetic relatedness among species, phylogenetic principal component analysis (pPCA) was performed to identify the dominant dimensions of root trait covariation using the “phytools” package (Revell, 2012). pPCA is an extended method of PCA that is a powerful multivariate analysis technique and can summarize a set of data on correlated variables with a few composite, uncorrelated principal components (James and McCulloch, 1990). pPCA has been widely used in the determination of independent axes of functional specialization (e.g., Wang et al., 2018a; Liu C. et al., 2019; Bergmann et al., 2020; Weigelt et al., 2021). The Kaiser’s eigenvalue greater than 1 rule was used to determine the intrinsic dimensionality of root trait covariation, this is, eigenvalues of the principal components greater than 1 were considered significant (Kaiser, 1958; Tabachnik and Fidell, 1996). This rule was used in previous studies that examined the main axes of plant trait covariation (e.g., Comas and Eissenstat, 2009; Chen et al., 2013; Jager et al., 2015; Kramer-Walter et al., 2016). To test whether the pattern of root trait covariation for all species was similar to those for different plant growth forms, mycorrhizal types, and biomes, the pPCA analysis was further repeated for subsets of plant growth forms (herbaceous and woody), mycorrhizal types (AM and ECM), and biomes (tropical, subtropical, temperate forest types, and alpine tundra). ERM, AM-ECM, AM-NM, boreal forest, and grassland did not perform the pPCA analysis as their sample sizes were small (*n* ≤ 15).

To assess the segregation of plant species by their phylogenetic clades, plant growth forms, mycorrhizal types, and biomes along the dominant axes of root trait covariation, one-way ANOVA was used on the scores of species on the pPCA axes and the significance of pairwise differences was tested using Tukey’s HSD *post hoc* test.

The variance partitioning analysis was used to determine the relative contributions of plant growth form, mycorrhizal type, and biome according to the pPCA scores of the dominant principal components of root trait covariation for all species. The significances of each factor and their interactions were tested using 999 permutations. The analysis was followed by Legendre and Legendre (2012) using the “vegan” package.

All statistical analyses were conducted using R version 4.0.3 (R Core Team, 2020).

## Results

### Root Trait Covariation

**FIGURE 1:**
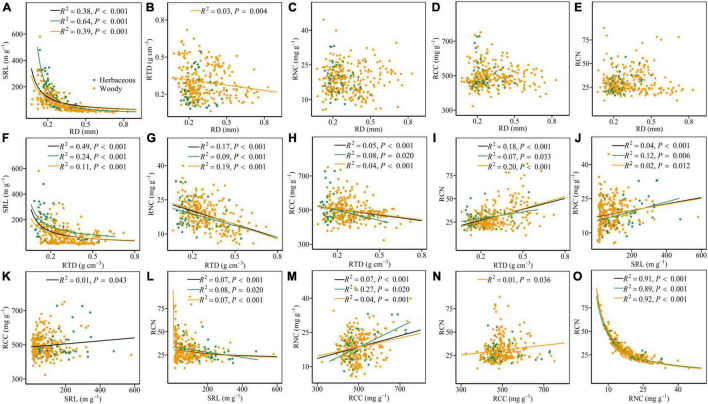
Pairwise relationships of the six root traits for all species (black) and within herbaceous (green) and woody species (yellow) **(A–O)**. The *R*^2^ (coefficient of determination) and *P*-values are obtained from the linear and non-linear regression analyses. RD, root diameter; SRL, specific root length; RTD, root tissue density; RNC, root N content; RCC, root C content; RCN, root C:N ratio.

For all species, RD was negatively associated with SRL (Figure 1A and Table 1). RD was not significantly associated with RTD, RNC, RCC, and RCN (Figures 1B–E), but it was negatively associated with RTD after considering the phylogenetic information (Table 1). Regardless of the phylogenetic relatedness among species being accounted for, SRL was always negatively and positively associated with RTD and RNC, respectively (Figures 1F,J). RTD, SRL, and RNC were significantly related to RCN (Figures 1I,L,O). However, RCC did not show a significant relationship with RCN (Figure 1N) but it showed significant relationships with RTD, SRL, and RNC (Figures 1H,K,M).

**TABLE 1:** Correlation coefficient matrix of the six root traits using Pearson’s correlation analysis (lower-left diagonal) and phylogenetic independent contrasts (PICs, upper-right diagonal) for all species.

	RD	SRL	RTD	RCC	RNC	RCN
RD		−0.55***	−0.33***	0.17***	0.04	0.23
SRL	−0.81***		−0.40***	−0.01	0.09	−0.10
RTD	−0.11	−0.39***		−0.43***	−0.40***	−0.23***
RCC	−0.01	0.11	−0.20***		0.66***	−0.12*
RNC	−0.02	0.26***	−0.43***	0.21***		−0.82***
RCN	0.02	−0.22***	0.36***	0.14*	−0.94***	

Root traits data are log_10_-transformed. The bold indicates that the correlations are significant: ***P < 0.001; *P < 0.05. RD, root diameter; SRL, specific root length; RTD, root tissue density; RCC, root C content; RNC, root N content; RCN, root C:N ratio.

**FIGURE 2:**
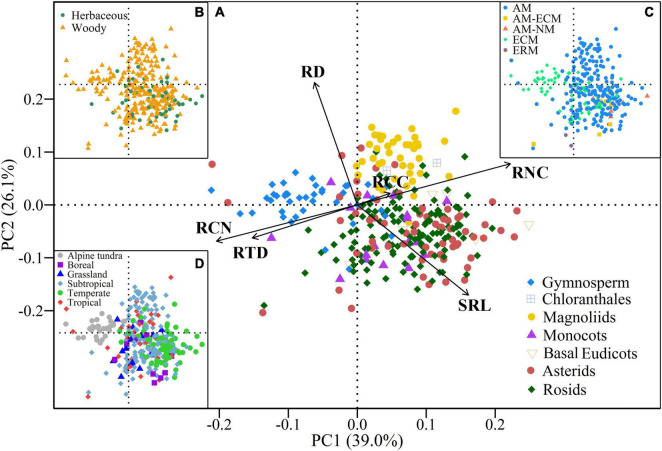
Phylogenetic principal component analysis (pPCA) results of the six root traits for all species. **(A–D)** pPCA results for all species coded by phylogenetic clades, plant growth forms, mycorrhizal types, and biomes, respectively. RD, root diameter; SRL, specific root length; RTD, root tissue density; RCC, root C content; RNC, root N content; RCN, root C:N ratio; AM, arbuscular mycorrhizal; ECM, ectomycorrhizal; ERM, ericoid mycorrhizal; NM, non-mycorrhizal.

All root traits (except RCC) exhibited significant phylogenetic signals (Table 2). The pPCA results showed that eigenvalues of the first three axes were greater than 1 (Table 2), indicating that the covariation in root traits was represented by three independent dimensions. The first three primary axes accounted for 85.3% of the total variation of root traits. The first PCA axis (PC1) accounted for 39.0% of the total variation and was mainly related to SRL, RTD, RNC, and RCN (Figure 2A). With increasing scores of the PC1 axis, SRL and RNC increased while RTD and RCN decreased. The second PCA axis (PC2) explained 26.1% of the total variation and showed a negative relationship between RD and SRL. The third PCA axis (PC3) accounted for an additional 20.2% of the total variation and was primarily associated with RCC. The species distribution within the trait space showed that gymnosperms had the lowest PC1 scores with higher RTD and RCN values. Magnoliids species had the highest PC2 values with higher RD and lower SRL values whereas monocots and eudicots species had lower PC2 values with lower RD and higher SRL values (Figure 2A and Supplementary Figure 1).

**TABLE 2:** Phylogenetic principal component analysis (pPCA) results and phylogenetic signals of the six root traits for all species.

	PC1	PC2	PC3	Blomberg’s *K*	*P*-value	Pagel’s λ	*P*-value
Eigenvalue	1.53	1.25	1.10				
Variation explained (%)	39.02	26.08	20.22				
RD	–0.25	**0.93**	0.10	0.12	0.001	0.78	<0.001
SRL	**0.65**	**−−0.68**	0.16	0.13	0.001	0.72	<0.001
RTD	**−−0.61**	**−−0.25**	–0.43	0.04	0.001	0.67	<0.001
RCC	0.19	0.09	**0.86**	0.01	0.976	<0.001	1.000
RNC	**0.89**	0.31	–0.11	0.03	0.029	0.68	<0.001
RCN	**−−0.82**	–0.27	0.49	0.04	0.001	0.65	<0.001
PC1				0.04	0.002	0.67	<0.001
PC2				0.07	0.001	0.79	<0.001
PC3				0.01	0.533	0.27	<0.001

*Root traits are log_10_-transformed. Bold indicates the variable loading scores with the greatest load on each component.*

*RD, root diameter; SRL, specific root length; RTD, root tissue density; RCC, root C content; RNC, root N content; RCN, root C:N ratio; PC1, PC2, and PC3 correspond to the first three main axes of the covariation of root traits based on the pPCA results.*

### Root Trait Covariation Across Plant Growth Forms, Mycorrhizal Types, and Biomes

**FIGURE 3:**
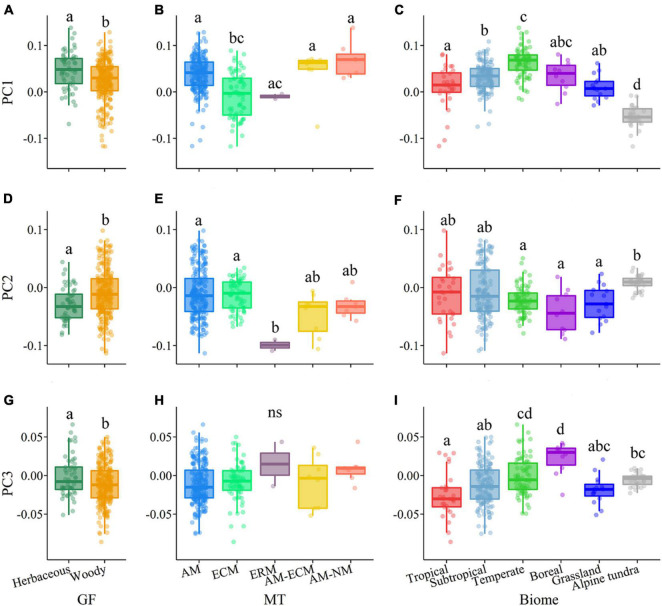
Distribution of plant growth forms, mycorrhizal types, and biomes along the first three main principal components (i.e., PC1–PC3) of the root trait covariation. **(A,D,G)** Plant growth form (GF); **(B,E,H)** mycorrhizal type (MT); **(C,F,I)** biomes. Letters represent statistically significant differences in the average PCs (Tukey’s HSD *post hoc* test, *P* < 0.05), such that groups not containing the same letter are different. In the box plots the central line represents the mean; the lower and upper box limits correspond to the 25th and 75th percentiles and the upper (lower) whiskers extend to 1.5 (–1.5) times the interquartile range, respectively. AM, arbuscular mycorrhizal; ECM, ectomycorrhizal; ERM, ericoid mycorrhizal; NM, non-mycorrhizal.

In terms of plant growth form, RD was negatively related to RTD in woody species, but this relationship was not observed in herbaceous species (Figure 1B and Supplementary Figures 2, 3). Compared to the herbaceous group, the woody group exhibited weaker relationships between SRL and RTD and RNC (Figures 1F,G). The ANOVA results showed that herbaceous and woody species showed significant differences in the scores of the first three axes of root trait covariation (i.e., PC1–PC3) (*P* < 0.05, Figures 3A,D,G). Herbaceous species had higher scores of the PC1 (e.g., higher SRL and RNC) and the lower scores of the PC2 (e.g., lower RD and higher SRL) compared with woody species. Woody species showed the consistent root trait covariation with the pattern of root trait covariation for all species, representing by three independent dimensions (Supplementary Table 1 and Supplementary Figure 4B). For herbaceous species, the first three axes explained 87.6% of the total variation in root traits (Supplementary Table 1 and Supplementary Figure 4A). Within the first axes, the main difference between herbaceous and woody species was that RD was strongly loaded on PC1 for herbaceous species. In addition, the second dimension was represented by RD, SRL, and RNC; the third dimension was dominated by RCC and RCN for herbaceous species.

The relationships among root traits differed across mycorrhizal types (Supplementary Figures 2, 3, 5). RD was negatively associated with RTD in AM species but was positively associated with RTD in ECM species (Supplementary Figure 5B). RD was negatively related to RNC in ECM species but showed no correlation in AM species (Supplementary Figure 5C). Notably, most correlations among root traits were stronger in ECM species than those in AM species. The ANOVA results showed that AM and ECM species showed significant differences in the scores of the PC1 but not for the PC2 and PC3 (Figures 3B,E,H). AM species had higher PC1 scores (i.e., higher SRL and RNC values) compared with ECM species. The AM group also showed three independent dimensions of root trait covariation, which was consistent with the pattern for all species (Supplementary Table 1 and Supplementary Figure 4C). ECM species also showed three independent axes of root trait variation. The PC1 axis was dominated by RD, RTD, RCN, SRL, and RNC, the PC2 axis was dominated by RCC, and the PC3 axis was dominated by RD, SRL, and RCN (Supplementary Table 1 and Supplementary Figure 4D).

Moreover, the correlations among root traits differed across biomes (Supplementary Figures 2, 3, 6). RD was negatively correlated with RTD in the tropical and subtropical forests, but not in the other biomes. SRL and RNC values decreased with increasing RTD only in the temperate forest. In addition, there were significant differences in the scores of PC1, PC2, and PC3 among biomes (*P* < 0.05, Figures 3C,F,I) and had different trait syndromes among different biomes (Figure 2D). Species in the temperate forest had the highest PC1 scores with higher SRL and RNC values, whereas species in the alpine tundra had the lowest PC1 scores with higher RCN and RTD values. Species in the tropical and subtropical forests showed higher variations on the PC2 axis compared with other biomes. The dominant dimensions of the covariation in root traits differed greatly across biomes. In the tropical and subtropical forests, the first three axes explained 90.0 and 86.5% of the total variation in root traits, respectively (Supplementary Table 2 and Supplementary Figures 4E,F). In these two forests, the PC1 axis was heavily loaded by RD, RTD, RNC, and RCN, and the PC2 axis was strongly represented by the negative relationships between RD and SRL. In addition, two independent root trait dimensions – representing root morphological traits (i.e., RD, RTD, and SRL) and nutrient traits (i.e., RNC and RCN) – were detected in the alpine tundra (Supplementary Table 2 and Supplementary Figure 4H).

### Relative Effects of Plant Growth Form, Mycorrhizal Type, and Biome on the Root Trait Covariation

**FIGURE 4:**
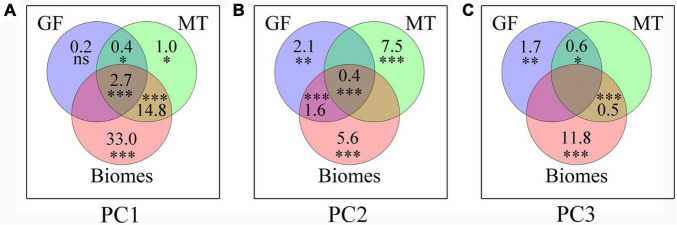
Relative contributions (%) of plant growth form, mycorrhizal type, and biome to the first three main principal components (i.e., PC1–PC3) of root trait covariation. The intersections represent variation that is jointly explained by two or more variable categories. **(A)** PC1; **(B)** PC2; **(C)** PC3. The number (lower-right) indicates the variations that are unexplained proportion by these three groups. The significances of each category are tested using 999 permutations. ^***^P < 0.001; ^**^P < 0.01; *P < 0.05; *^ns^P* > 0.05. GF, plant growth form; MT, mycorrhizal type.

Plant growth form, mycorrhizal type, and biome together accounted for 52.0, 14.1, and 13.8% of the total variation of PC1, PC2, and PC3, respectively (Figure 4). The PC1 axis was mostly explained by biome alone (33.0%), followed by the interactive effects of biome and mycorrhizal type (14.8%). The PC2 axis was mostly explained by mycorrhizal type alone (7.5%) and biome alone (5.6%), and the PC3 axis was mainly influenced by biome alone (11.8%), followed by plant growth form alone (1.7%).

## Discussion

### Multidimensional Pattern of Variation in Root Traits

Our study demonstrated three independent dimensions of the covariation among the six root traits in China. The first dimension was dominated by SRL, RTD, RNC, and RCN that were closely interrelated, which is in accord with the RES expectations. This result is not in agreement with the previous studies reporting that RD and SRL were orthogonal to RTD and RNC (Kramer-Walter et al., 2016; Wang et al., 2018a; McCormack and Iversen, 2019; Bergmann et al., 2020). Within the first axis, in one end, species had high RTD, high RCN, low SRL, and low RNC associated with a resource-conservation strategy, whereas in the other end species had high SRL, high RNC, low RTD, and low RCN associated with a resource-acquisitive strategy (Roumet et al., 2016; de la Riva et al., 2018). As the expected from the RES, we found that SRL was negatively associated with RTD and positively related to RNC, suggesting a trade-off between resource acquisition and construction costs of roots (Makita et al., 2012; Li et al., 2019). According to the RES expectations, fine roots with a higher SRL should be associated with a higher uptake activity and metabolic rate (i.e., higher RNC) and shorter lifespan (i.e., lower RTD) to maximize resource acquisition per investment (Eissenstat et al., 2000; Weemstra et al., 2016; de la Riva et al., 2021b). RD was not related to RNC, RTD, and RCN that strongly loaded on the first axis, thus RD was decoupled from the first axis. In addition, the second axis was dominated by a trade-off between RD and SRL, which did not support the previous studies reporting that the variation of root traits was mostly explained by RD and SRL (Wang et al., 2018a; McCormack and Iversen, 2019; Bergmann et al., 2020). Recent studies have reported that RD and SRL were positively and negatively related to the percentage of mycorrhizal colonization, respectively (Ma et al., 2018; Bergmann et al., 2020), suggesting that roots can enhance resource uptake from soils by constructing either thin-diameter roots with higher SRLs (i.e., “do-it-yourself” strategy), or in contrast thick-diameter roots *via* reliance on mycorrhizal associations (i.e., “outsourcing” strategy) (McCormack and Iversen, 2019; Bergmann et al., 2020). However, roots with a larger mycorrhizal colonization may increase the resource uptake capacity under resource-limited conditions without necessarily implying an acquisitive strategy (Navarro-Fernández et al., 2016). Therefore, it is needed to uncover how this root–mycorrhizal collaboration gradient links to the differences in resource uptake capacity for species with fast- and slow-traits (de la Riva et al., 2021a). Furthermore, we found that RCC formed an additional axis that was decoupled from the first two dimensions. This lack of correlation may be attributable to two reasons. First, root traits that were related to the first two axes displayed significant phylogenetic conservatism, but RCC did not, suggesting that these trait associations are not tightly coupled. Second, in theory, RCC, RNC, and RCN are mathematically interdependent. However, we observed that RNC was strongly associated with RCN, whereas RCC was not. This indicated that RNC, rather than RCC, was more important in the trade-offs between C investment and resource uptake in roots (An et al., 2021). Our results is not consistent with the global and regional studies (Kramer-Walter et al., 2016; Wang et al., 2018a; McCormack and Iversen, 2019; Bergmann et al., 2020), we found that the RES explained the most variation of root traits and the trade-off between RD and SRL loaded on the second axis in China, suggesting that root trait covariation may not be generalized from global-scale and other regional analyses.

**FIGURE 5:**
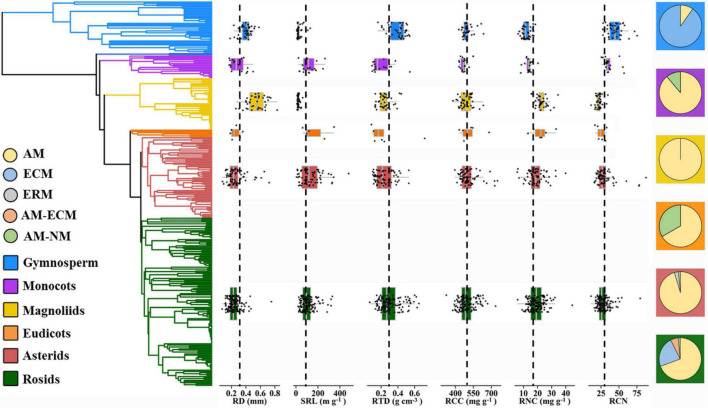
Trait distribution for RD, SRL, RTD, RCC, RNC, RCN, and mycorrhizal association for six major phylogenetic clades (gymnosperms, monocots, magnoliids, basal eudicots, asterids, and rosids). Basal eudicots include species that are not included in the clades of rosids and asterids. Chloranthales is not included because the species number is small (*n* = 2). The left indicates the phylogenetic tree of 306 species. Dashed line along the box graphs (middle) represents the arithmetic mean of each root trait. Pie charts (right) indicate the proportion of each mycorrhizal type in each phylogenetic clade. RD, root diameter; SRL, specific root length; RTD, root tissue density; RCC, root C content; RNC, root N content; RCN, root C:N ratio; AM, arbuscular mycorrhizal; ECM, ectomycorrhizal; ERM, ericoid mycorrhizal; NM, non-mycorrhizal.

Furthermore, our results revealed that the pattern of variation in root traits was closely linked to the phylogenetic structure; that is, species within different phylogenetic clades occupied different locations in the trait space. Gymnosperms generally dominated boreal and subalpine forests that are mainly constrained by low temperatures, as thick roots with high RTD values tend to have high physical robustness to cope with cold environments (Simpson et al., 2016; Wang et al., 2018b; Yahara et al., 2019). Early diverged magnoliids are generally associated with phosphorus-limited tropical and subtropical soils (Ma et al., 2018), resulting in high RD and RNC values (Figure 5). Thick roots maximize the cortex area, supporting more AM colonization as a complementary strategy for nutrient foraging, leading to high RNC (Brundrett, 2002; Comas et al., 2012; Kong et al., 2019). Moreover, recently diverged eudicots species were more concentrated toward lower RD and higher SRL values. This can allow roots to increase their surface area and explore larger soil volumes per unit of C investment – that is, a “do-it-yourself” strategy (Comas et al., 2012; Valverde-Barrantes et al., 2017; Bergmann et al., 2020).

### The Drivers of the Covariation Among Root Traits

#### Biome

Our study showed that biome type was the most important factor influencing the variation of the three independent dimensions, suggesting that roots have evolved contrasting resource acquisition strategies among different biomes. In addition, we observed that the correlations among root traits varied across biomes. This result was likely due to the differences in environmental constraints (Ostonen et al., 2017) and species composition across biomes (Roumet et al., 2016). Differences in environmental condition and phylogenetic group may cause differences in root trait adjustment (Wang et al., 2018b) and mycorrhizal dependency (Valverde-Barrantes et al., 2016), and changes in both factors could strengthen, weaken, or fully decouple correlations among root traits (Ma et al., 2018; Valverde-Barrantes et al., 2020, 2021). For example, RD was negatively associated with RTD in the tropical and subtropical forests, which is consistent with the study of Valverde-Barrantes et al. (2021). Such a negative relationship can be explained by the anatomical relationships that is commonly observed in leaves (Laughlin, 2014; de la Riva et al., 2016a), plants can have similar specific leaf area (or SRL) values with different proportional investments in leaf tissue density (or RTD) and leaf thickness (or RD) (John et al., 2017; de la Riva et al., 2021a), which would depend on the species identity and their environmental conditions (Olmo et al., 2014). We found that RTD was negatively related to SRL in the temperate forest, suggesting the trade-off between resource acquisition and construction costs of roots (Eissenstat et al., 2000). From the cost-benefit theory, roots with higher SRL and lower RTD would have lower construction costs, higher metabolic rates and faster return of investments (de la Riva et al., 2021b). Furthermore, species in the alpine tundra tended to have more conservative strategies with higher RTD, lower SRL, and RNC, which may be attributable to the ECM-dominated gymnosperms in the alpine tundra in our study. Some root morphological adaptations, such as low cortex area and high branching intensity, are achieved before shifts from AM to alternative mycorrhizal associations (Comas and Eissenstat, 2009; Valverde-Barrantes et al., 2016, 2018). Thus, species associated with ECM fungi were less dependent on the root cortex, and thicker roots with highly lignified stele tissues were closely linked to higher RTD (Guo et al., 2008; Kong et al., 2019). These comparisons among plants in widely disparate biomes from China provide the basis for predicting differences in root trait trade-offs between biomes.

#### Mycorrhizal Type

Mycorrhizal type also played an important role in the three main dimensions of root trait variation, especially for the PC2 axis. This result provides new evidence for root–mycorrhizal collaboration gradient representing tradeoffs between “do-it-yourself” and “outsourcing” for resource uptake (McCormack and Iversen, 2019; Bergmann et al., 2020). In agreement with previous studies (Comas et al., 2014; Valverde-Barrantes et al., 2018; de la Riva et al., 2021b), AM species tended to have more acquisitive strategies with higher SRL and lower RTD while ECM species were associated with more conservative strategies with lower SRL and higher RTD within the PC1 axis. This may be due to the differences in anatomical and morphological adaption between AM and ECM species. Species colonized by AM fungi exhibited a rapid resource uptake strategy with a higher investment in root length per unit root mass (i.e., higher SRL) (Guo et al., 2008; Valverde-Barrantes et al., 2018; Kong et al., 2019). In addition, the first axis was mostly driven by the RES for both AM and ECM species (38.3 and 47.2% of the variance, respectively). However, compared with AM species, ECM species showed a more consistent pattern of the RES because RD was included in the first axis, in which RD and RTD were negatively correlated with SRL, RNC, and RCN. The difference between these two mycorrhizal types may be related to the lower number of species in ECM group compared with AM group. In addition, we found that RD had different associations with RTD and RNC between AM and ECM groups. Among ECM species, RD showed positive and negative relationships with RTD and RNC, respectively, which is in accord with the RES hypothesis (Freschet et al., 2010; Reich, 2014) and also supports the study of Kong et al. (2019) at the global scale. Such relationships in our study could be explained by the typical features of nutrient acquisition in ECM species. ECM species predominately form Hartig nets in the intercellular spaces of root tips and are less dependent on cortex tissue (Brundrett, 2002; Comas et al., 2014), leading to positive correlations between RD and RTD. ECM plants with thin roots have a thick fungal mantle that is relatively rich in N and enhances the N content of thin roots compared to that of thick roots (Kong et al., 2019). Our study demonstrated that AM and ECM species had contrasting root traits syndromes, however, the pattern of root trait variation in these two mycorrhizal types was mostly driven by the RES. These results suggested that the differences in resource acquisition strategies between them were not only determined by the type of mycorrhizal association. Therefore, further studies should include direct measurements of mycorrhizal colonization (e.g., percentage of mycorrhizal colonization) to confirm the collaboration gradient proposed in the global studies and investigate the trade-offs between root acquisition and conservation in relation to the symbiotic roles presented here.

#### Plant Growth Form

We found that plant growth form had a weak effect on the three main axes of root trait variation. Our results are consistent with previous studies reporting that herbaceous species have more acquisitive strategies with thinner RD, higher SRL and RNC compared with woody species (Valverde-Barrantes et al., 2017). In addition, the first axis was dominated by the RES and the second axis was dominated by RD and SRL for both herbaceous and woody species. However, the main difference between these two plant growth forms was that RD was included in the first axis for herbaceous species, leading to herbaceous species with a more consistent pattern with the RES compared with woody species. Such discrepancy between them may be related to two possible reasons. First, RD was negatively related to RTD for woody species, which is in line with previous studies reporting a trade-off between RD and RTD in Mediterranean woody plants (de la Riva et al., 2016b,2021a). As mentioned above, such a negative relationship can be explained by the anatomical trade-offs between RD and RTD. Second, Ma et al. (2018) has reported that woody species have approximately 30% more mycorrhizal colonization than herbaceous species for a given RD, suggesting that roots of herbaceous species have become less dependent on mycorrhizae fungi. In addition, herbaceous roots have evolved more efficient trait syndromes (e.g., thinner diameter, higher SRL, and lower RTD), which may change the relationships among root traits. Our results suggested the differences in root ecological strategies among plant growth forms, which can help elucidate the trade-offs between root construction and function and their influences on ecosystem functions.

### Uncertainties and Future Research Needs

The uncertainties of this study lie in four aspects due to the restrictions of data coverage. First, our study focused on commonly studied mycorrhizal statuses (i.e., AM and ECM), some other mycorrhizal types such as ERM, NM, and dual associations were not considered in our analyses owing to the limited number of root samples. The type of mycorrhizal partnership is an important driver of the variation in root traits and their functions (Valverde-Barrantes et al., 2017; Gao et al., 2021). More detailed studies of mycorrhizal status are needed to elucidate how the evolution of root traits and their mycorrhizal associations affect belowground processes in seed plants. Second, the uneven distribution of data across biomes is likely to impede our understanding of the effect of biomes on root resource uptake. In particular, we had a limited number of species in the boreal forest and grassland, which may explain the weak or insignificant relationships among root traits in these biomes. Further work with a wide representation of species from multiple biomes is crucial to improve our understanding of the role of biomes in resource acquisition strategies in fine-root systems. Third, our analysis focused on the interspecific variation in root traits, thus resulting in some uncertainties regarding the importance of intraspecific variation and plasticity in trait-based studies (Jung et al., 2010; Weemstra et al., 2020). Future studies that incorporate the intraspecific trait plasticity will help to elucidate the trade-offs among root traits related to belowground resource acquisition strategies (Isaac et al., 2017). Finally, we considered six root morphological and chemical traits in our study, however, the root trait covariation may be dependent on the trait variables studied. de la Riva et al. (2021a) demonstrated that SRA (i.e., mass-normalized) was more tightly correlated than SRL with the dry matter content and chemical composition of both roots and leaves along the economics spectrum for Mediterranean vegetation. Therefore, it is important to measure and integrate the root trait data based on the standardized approaches, which can be analogous to traits related to the LES, including root morphology (e.g., SRA and root dry matter content), root chemistry (e.g., RNC and lignin), root anatomy (e.g., cortical area and stele area), percentage of mycorrhizal colonization, and root function (e.g., respiration, decomposition, and resources uptake) (Laliberte, 2017). This would allow us to develop a more general integrated framework related to the trade-offs between root structure and function and their underlying mechanisms.

## Conclusion

Our study provides a comprehensive assessment of the covariation among root traits and their generality across plant growth forms, mycorrhizal types, and biomes using the largest root trait database in China. Three independent root trait dimensions were identified, where the first dimension was related to trade-offs between resource acquisition and conservation (i.e., SRL, RTD, RNC, and RCN) that was defined as the RES, the second dimension was related to RD and SRL, and the third dimension was dominated by RCC. Biome and mycorrhizal type were the most important factors in driving the variation of the three main dimensions. Furthermore, the root trait covariation was dependent on plant growth form, mycorrhizal type, and biome. More importantly, we found a more consistent pattern of the root trait variation related to the RES in herbaceous and ECM species compared with woody and AM species, indicating high coordination among root morphological and chemical traits in herbaceous and ECM species. These results demonstrate that the covariation among root traits was more complex across plant growth forms, mycorrhizal types, and biomes at the regional scale than those at the global scale, indicating a critical role of spatial scale in influencing the generality of associations among root traits. Further work on the covariation among root traits at different spatial scales will contribute to our understanding of plant form and function and help predict belowground responses to changing environmental conditions.

## Data Availability Statement

The raw data supporting the conclusions of this article will be made available by the authors, without undue reservation.

## Author Contributions

NA conceived the ideas and collected the data. NA and NL led the writing of the manuscript. All authors contributed critically to the drafts and gave the final approval for publication.

## Conflict of Interest

The authors declare that the research was conducted in the absence of any commercial or financial relationships that could be construed as a potential conflict of interest.

## Publisher’s Note

All claims expressed in this article are solely those of the authors and do not necessarily represent those of their affiliated organizations, or those of the publisher, the editors and the reviewers. Any product that may be evaluated in this article, or claim that may be made by its manufacturer, is not guaranteed or endorsed by the publisher.

## Footnotes

1
http://roots.ornl.gov

2
http://www.theplantlist.org/

3
https://www.try-db.org/TryWeb/Data.php#3
