# Supplementary material for: Evidence of Differences in Covariation Among Root Traits Across Plant Growth Forms, Mycorrhizal Types, and Biomes
Source: Front Plant Sci. 2022 Jan 28;12:785589. doi: 10.3389/fpls.2021.785589 (PMC8836870; doi:10.3389/fpls.2021.785589)
Supplement: Supplementary file 1 [file Data_Sheet_1.docx]

**Supplementary material**

**Evidence of differences in covariation among root traits across plant growth forms, mycorrhizal types, and biomes**

**Note S1** Besides for global Fine-Root Ecology Database (FRED, http://roots.ornl.gov), root trait data was extracted from published literature that were listed as follows.

Chen, G.T., Tu, L.H., Peng, Y., Hu, H.L., Hu, T.X., Xu, Z.F., Liu, L., Tang, Y., 2017. Effect of nitrogen additions on root morphology and chemistry in a subtropical bamboo forest. Plant Soil 412 (1-2), 441-451. https://doi.org/10.1007/s11104-016-3074-z.

Ding, J.X., Kong, D.L., Zhang, Z.L., Cai, Q., Xiao, J., Liu, Q., Yin, H.J., 2020. Climate and soil nutrients differentially drive multidimensional fine root traits in ectomycorrhizal-dominated alpine coniferous forests. J. Ecol. 108 (6), 2544-2556. https://doi.org/10.1111/1365-2745.13407.

Kong, D.L., Ma, C.G., Zhang, Q., Li, L., Chen, X.Y., Zeng, H., Guo, D.L., 2014. Leading dimensions in absorptive root trait variation across 96 subtropical forest species. New Phytol. 203 (3), 863-872. https://doi.org/10.1111/nph.12842.

Wang, J.J., Tharayil, N., Chow, A.T., Suseela, V., Zeng, H., 2015. Phenolic profile within the fine-root branching orders of an evergreen species highlights a disconnect in root tissue quality predicted by elemental- and molecular-level carbon composition. New Phytol. 206 (4), 1261-1273. https://doi.org/10.1111/nph.13385.

Wang, R.L., Wang, Q.F., Zhao, N., Xu, Z.W., Zhu, X.J., Jiao, C.C., Yu, G.R., He, N.P., 2018. Different phylogenetic and environmental controls of first-order root morphological and nutrient traits: evidence of multidimensional root traits. Funct. Ecol. 32 (1), 29-39. https://doi.org/10.1111/1365-2435.12983.

Xu, Y., Gu, J.C., Dong, X.Y., Liu, Y., Wang, Z.Q., 2011. Fine root morphology, anatomy and tissue nitrogen and carbon contents of the first five orders in four tropical hardwood species in Hainan Island, China. Acta Phytoecologica Sinica 35 (9), 955-964.

Zhou, M., Bai, W.M., Zhang, Y.S., Zhang, W.H., 2018. Multi-dimensional patterns of variation in root traits among coexisting herbaceous species in temperate steppes. J. Ecol. 106 (6), 2320-2331. https://doi.org/10.1111/1365-2745.12977.

**Table S1** Phylogenetic principal component analysis (pPCA) results for the six root traits among different plant growth forms and mycorrhizal types.

|  |  | **PC1** | **PC2** | **PC3** |
| --- | --- | --- | --- | --- |
|  | Eigenvalue | 1.66 | 1.16 | 1.06 |
|  | Variation (%) | 46.2 | 22.5 | 18.9 |
|  | RD | **0.61** | **0.68** | 0.15 |
| Herbaceous | SRL | **-0.83** | **-0.52** | 0.16 |
| N=53 | RTD | **0.66** | 0.00 | -0.45 |
| Figure S4a | RCC | -0.36 | 0.45 | **0.68** |
|  | RNC | **-0.80** | **0.54** | -0.20 |
|  | RCN | **0.70** | -0.35 | **0.61** |
|  | Eigenvalue | 1.50 | 1.28 | 1.11 |
|  | Variation (%) | 37.5 | 27.3 | 20.4 |
|  | RD | -0.12 | **0.95** | 0.09 |
| Woody | SRL | **0.53** | **-0.76** | 0.18 |
| N=253 | RTD | **-0.59** | -0.27 | -0.45 |
| Figure S4b | RCC | 0.14 | 0.03 | **0.87** |
|  | RNC | **0.92** | 0.20 | -0.09 |
|  | RCN | **-0.86** | -0.19 | 0.46 |
|  | Eigenvalue | 1.52 | 1.25 | 1.09 |
|  | Variation (%) | 38.3 | 26.0 | 20.0 |
|  | RD | -0.30 | **0.91** | 0.04 |
| AM | SRL | **0.65** | **-0.66** | 0.23 |
| N=221 | RTD | **-0.54** | -0.28 | -0.46 |
| Figure S4c | RCC | 0.20 | 0.21 | **0.80** |
|  | RNC | **0.88** | 0.34 | -0.15 |
|  | RCN | **-0.82** | -0.26 | 0.50 |
|  | Eigenvalue | 1.68 | 1.24 | 1.03 |
|  | Variation (%) | 47.2 | 25.4 | 17.7 |
|  | RD | **0.60** | -0.53 | **0.55** |
| ECM | SRL | **-0.86** | 0.10 | **-0.46** |
| N=68 | RTD | **0.59** | 0.65 | 0.07 |
| Figure S4d | RCC | -0.08 | **-0.86** | -0.28 |
|  | RNC | **-0.86** | -0.17 | 0.41 |
|  | RCN | **0.81** | -0.23 | **-0.54** |

Root traits are log_10_-transformed. Bold indicates the variable loading scores with the greatest load on each component. RD, root diameter; SRL, specific root length; RTD, root tissue density; RCC, root C content; RNC, root N content; RCN, root C:N ratio; AM, arbuscular mycorrhizal; ECM, ectomycorrhizal.

**Table S2** Phylogenetic principal component analysis results (pPCA) for the six root traits among different biomes.

|  |  | **PC1** | **PC2** | **PC3** |
| --- | --- | --- | --- | --- |
|  | Eigenvalue | 1.53 | 1.43 | 1.00 |
|  | Variation (%) | 38.9 | 34.3 | 16.8 |
|  | RD | **0.59** | **0.74** | -0.10 |
| Tropical forest | SRL | -0.31 | **-0.88** | 0.06 |
| N=31 | RTD | **-0.76** | -0.28 | -0.17 |
| Figure S4e | RCC | -0.33 | 0.20 | **-0.90** |
|  | RNC | **0.74** | -0.54 | -0.37 |
|  | RCN | **-0.80** | 0.57 | 0.09 |
|  | Eigenvalue | 1.45 | 1.28 | 1.20 |
|  | Variation (%) | 35.2 | 27.2 | 24.1 |
|  | RD | **-0.54** | **0.73** | 0.21 |
| Subtropical forest | SRL | 0.30 | **-0.61** | **-0.68** |
| N=144 | RTD | **0.38** | -0.29 | **0.73** |
| Figure S4f | RCC | 0.02 | 0.57 | **-0.61** |
|  | RNC | **-0.91** | -0.28 | -0.15 |
|  | RCN | **0.86** | 0.49 | -0.09 |
|  | Eigenvalue | 1.55 | 1.26 | 1.04 |
|  | Variation (%) | 40.3 | 26.6 | 18.2 |
|  | RD | **-0.65** | **0.69** | 0.14 |
| Temperate forest | SRL | **0.76** | -0.51 | 0.35 |
| N=77 | RTD | -0.25 | -0.28 | **-0.90** |
| Figure S4g | RCC | 0.34 | **0.70** | -0.02 |
|  | RNC | **0.81** | 0.53 | -0.24 |
|  | RCN | **-0.77** | -0.12 | 0.27 |
|  | Eigenvalue | 1.64 | 1.33 | 0.94 |
|  | Variation (%) | 44.7 | 29.4 | 14.8 |
|  | RD | -0.47 | **0.85** | 0.03 |
| Alpine tundra | SRL | 0.68 | **-0.63** | -0.17 |
| N=29 | RTD | -0.36 | **-0.68** | 0.34 |
| Figure S4h | RCC | 0.45 | 0.16 | **0.85** |
|  | RNC | **0.92** | 0.28 | -0.01 |
|  | RCN | **-0.90** | -0.28 | 0.14 |

Root traits are log_10_-transformed. Bold indicates the variable loading scores with the greatest load on each component. RD, root diameter; SRL, specific root length; RTD, root tissue density; RCC, root C content; RNC, root N content; RCN, root C:N ratio.


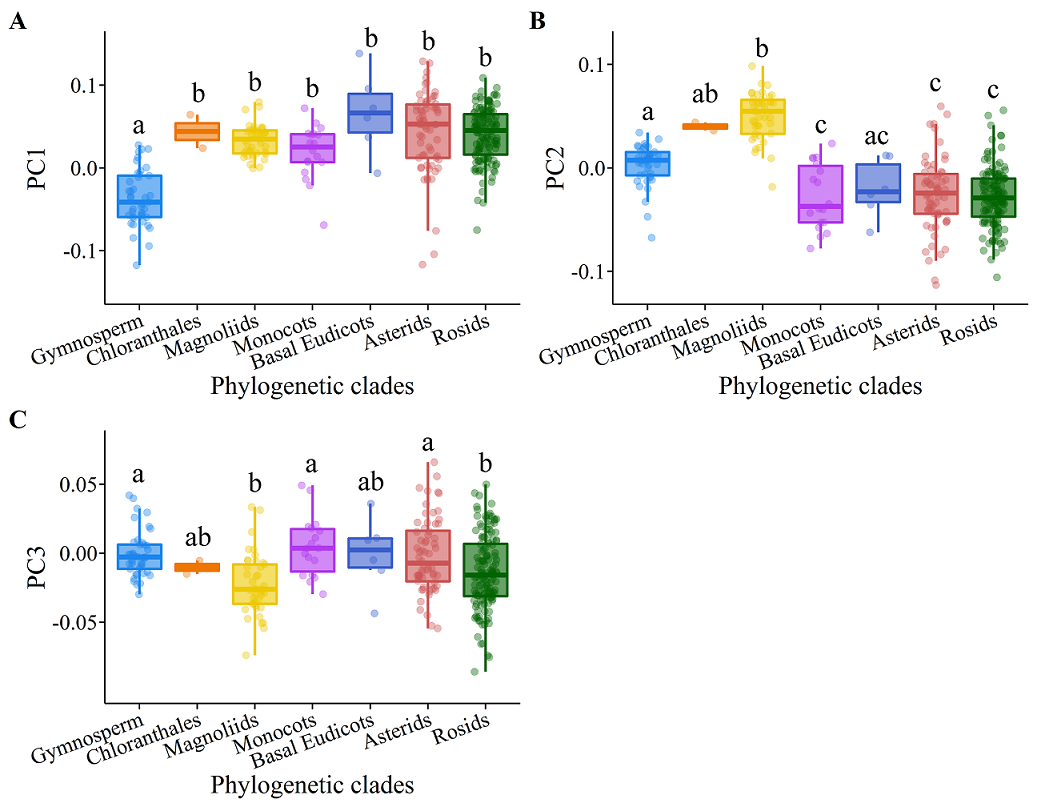


**Figure S1** Distribution of phylogenetic clades along the first three main principal components (i.e., PC1-PC3). Letters represent statistically significant differences in the average PCs (Tukey’s HSD test, *P*  <  0.05), such that groups not containing the same letter are different. In the box plots the central line represents the mean values; the lower and upper box limits represent the 25th and 75th percentiles and the upper (lower) whiskers extend to 1.5 (-1.5) times the interquartile range, respectively.

**
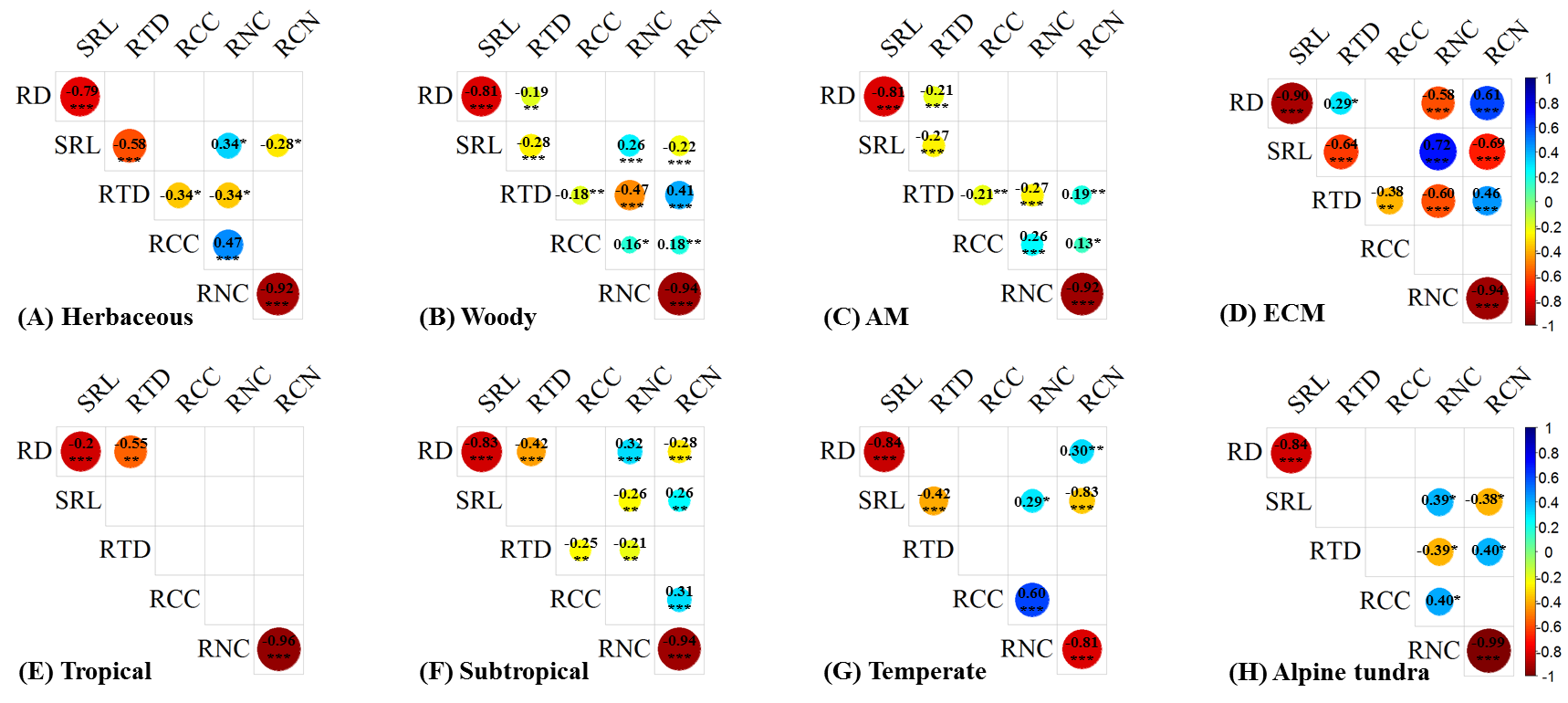
**

**Figure S2** Correlations among root traits for different plant growth forms **(A-B)**, mycorrhizal types **(C-D)**, and biomes **(E-H)** using Pearson’s correlation analysis. Asterisks indicate that the correlations are significant: ***, *P* < 0.001; **, *P* < 0.01; *, *P* < 0.05. The blank indicates that the correlations are not significant (*P* > 0.05). The size of the circles is proportional to the correlation coefficient. Root trait data are log_10_-transformed. RD, root diameter; SRL, specific root length; RTD, root tissue density; RCC, root C content; RNC, root N content; RCN, root C:N ratio; AM, arbuscular mycorrhizal; ECM, ectomycorrhizal.


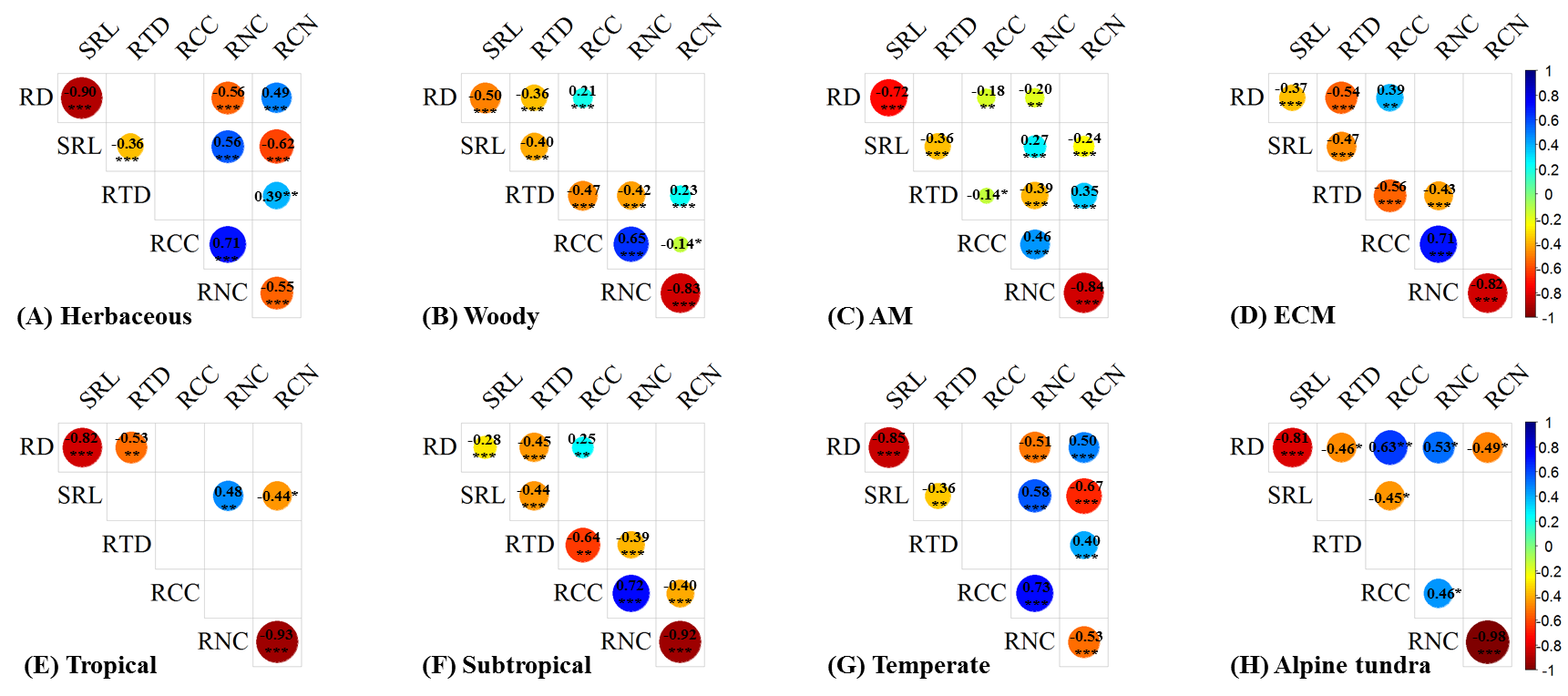


**Figure S3** Correlations among root traits for different plant growth forms **(A-B)**, mycorrhizal types **(C-D)**, and biomes **(E-H)** using phylogenetic independent contrasts (PICs). Asterisks indicate that the correlations are significant: ***, *P* < 0.001; **, *P* < 0.01; *, *P* < 0.05. The blank indicates that the correlations are not significant (*P* > 0.05). The size of the circles is proportional to the correlation coefficient. Root trait data are log_10_-transformed. RD, root diameter; SRL, specific root length; RTD, root tissue density; RCC, root C content; RNC, root N content; RCN, root C:N ratio; AM, arbuscular mycorrhizal; ECM, ectomycorrhizal.


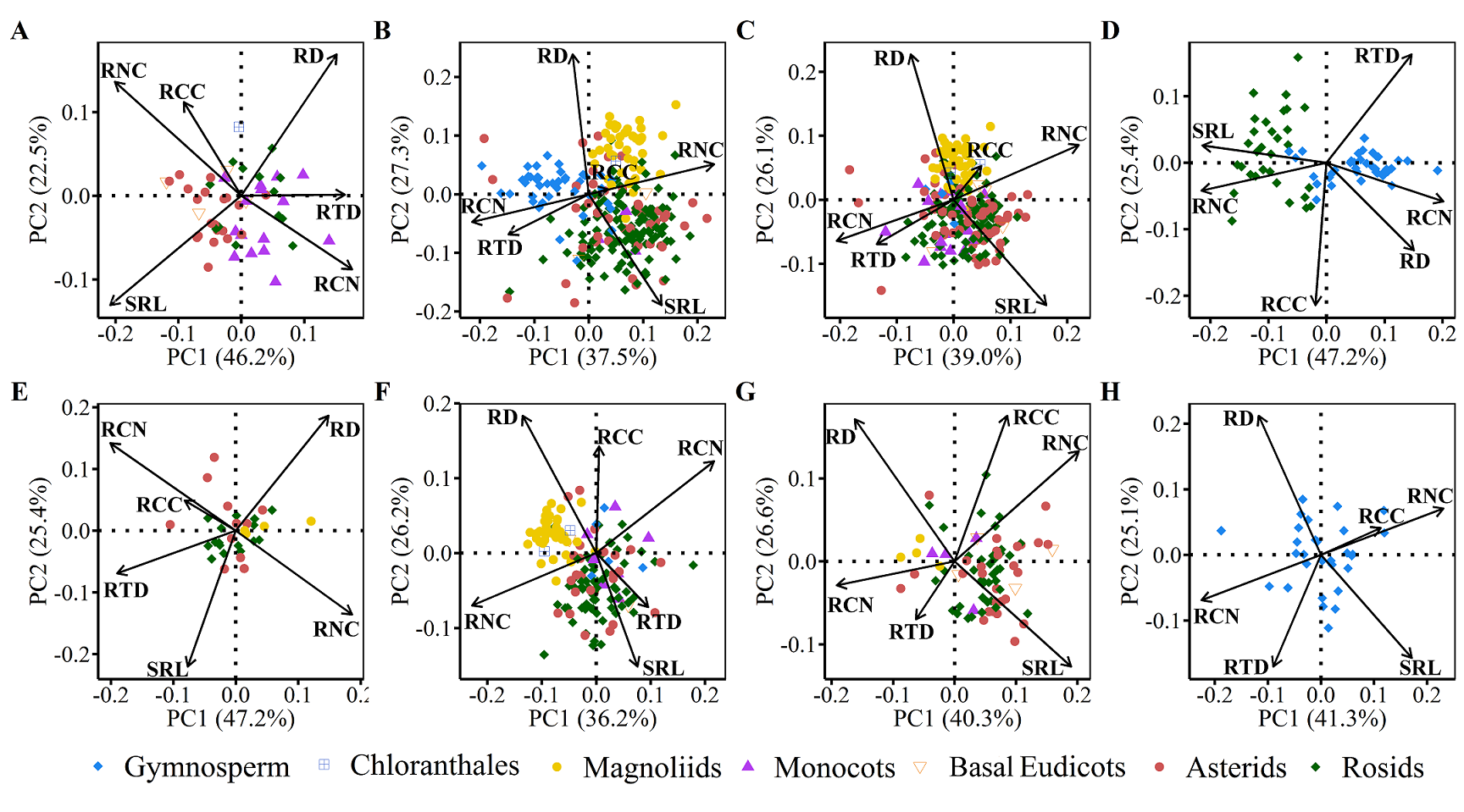


**Figure S4** Phylogenetic principal component analysis (pPCA) results of the six root traits across plant growth forms, mycorrhizal types, and biomes. **A**, herbaceous; **B**, woody; **C**, AM; **D**, ECM; **E**, tropical forest; **F**, subtropical forest; **G**, temperate forest; **H**, alpine tundra. AM, arbuscular mycorrhizal; ECM, ectomycorrhizal; RD, root diameter; SRL, specific root length; RTD, root tissue density; RCC, root C content; RNC, root N content; RCN, root C:N ratio; AM, arbuscular mycorrhizal; ECM, ectomycorrhizal.

**
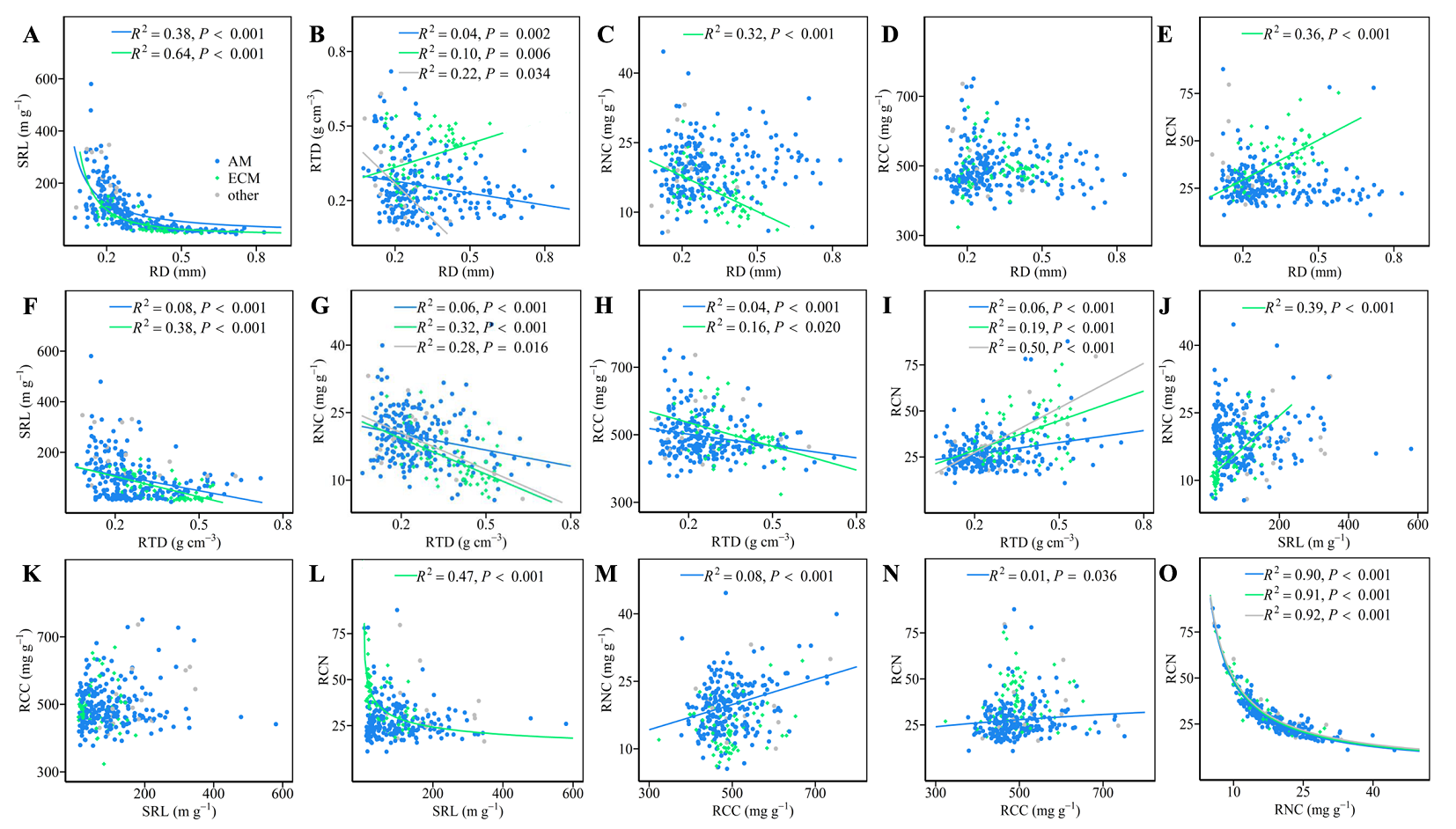
**

**Figure S5** Pairwise relationships of the six root traits across mycorrhizal types. The R^2^ (coefficient of determination) and *P*-values are obtained from the linear and nonlinear regression analyses. AM, arbuscular mycorrhizal (blue); ECM, ectomycorrhizal (green); Others, mycorrhizal types except for AM and ECM (grey). RD, root diameter; SRL, specific root length; RTD, root tissue density; RCC, root C content; RNC, root N content; RCN, root C:N ratio.

**
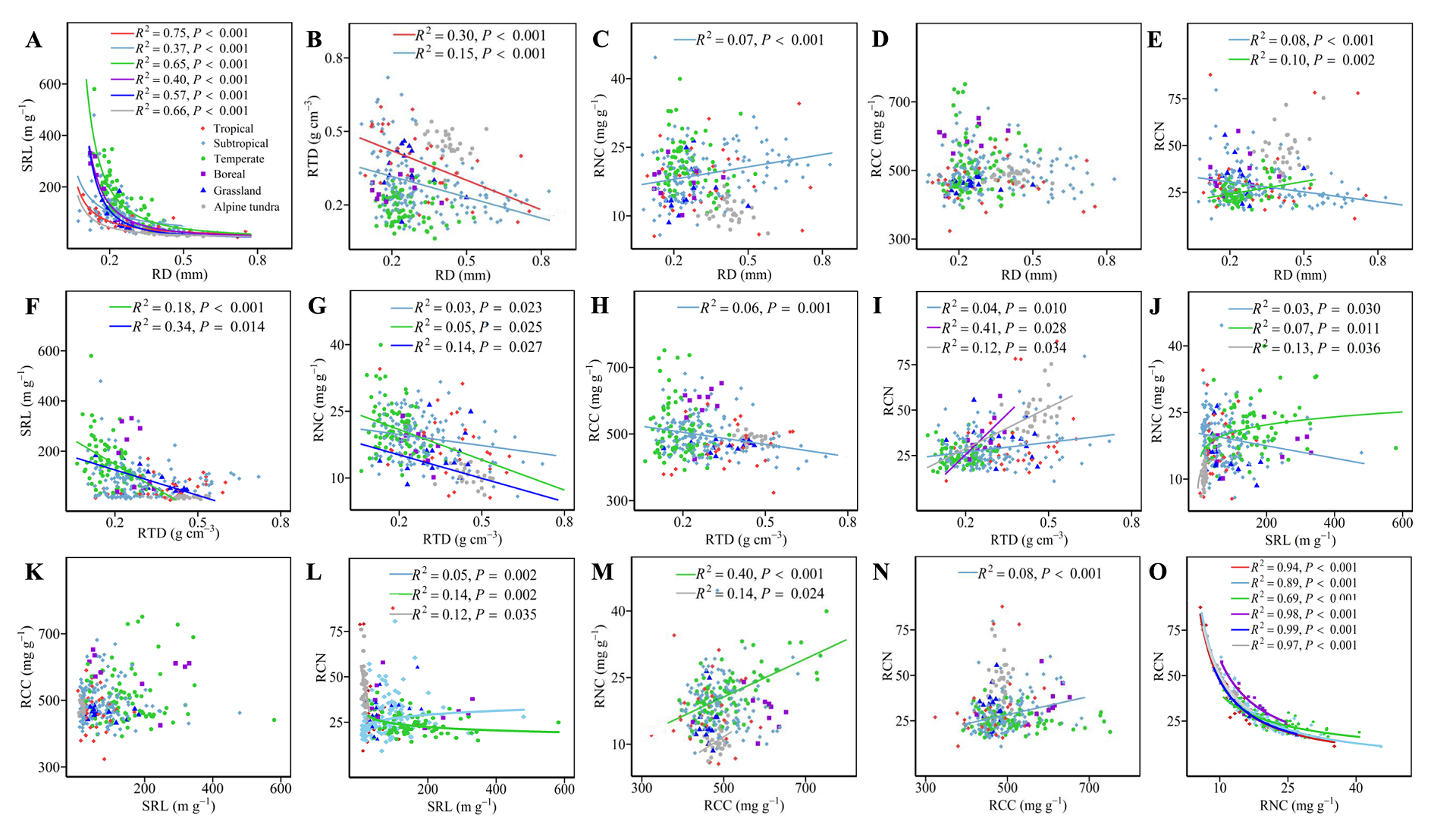
**

**Figure S6** Pairwise relationships of the six root traits across biomes. The R^2^ (coefficient of determination) and *P*-values are obtained from the linear and nonlinear regression analyses. Tropical forest (red); subtropical forest (light blue); temperate forest (green); boreal forest (purple); grassland (dark blue); alpine tundra (grey). RD, root diameter; SRL, specific root length; RTD, root tissue density; RCC, root C content; RNC, root N content; RCN, root C:N ratio.
